# Barriers and facilitators to integrated cancer care between primary and secondary care: a scoping review

**DOI:** 10.1007/s00520-023-08278-1

**Published:** 2024-01-22

**Authors:** Nicole Collaço, Kate A. Lippiett, David Wright, Hazel Brodie, Jane Winter, Alison Richardson, Claire Foster

**Affiliations:** 1 Centre for Psychosocial Research in Cancer (CentRIC+), School of Health Sciences, University of Southampton, Southampton, SO17 1BJ England; 2 School of Health Sciences, University of Southampton, Southampton, England; 3 Wessex Cancer Alliance, Oakley Road, Southampton, England; 4 University Hospital Southampton NHS Foundation Trust, Southampton, England

**Keywords:** Integrated care, Personalised care, Cancer, Models of care, Scoping review, Health services research

## Abstract

**Purpose:**

This scoping review identifies and characterises reported barriers and facilitators to providing integrated cancer care reported in the international literature, and develops recommendations for clinical practice.

**Methods:**

This scoping review included literature published between 2009 and 2022 and describes the delivery of integrated cancer care between primary and secondary care sectors. Searches were conducted of an online database Ovid Medline and grey literature.

**Results:**

The review included thirty-two papers. Barriers and facilitators to integrated cancer care were identified in three core areas: (1) at an individual user level around patient-healthcare professional interactions, (2) at an organisational level, and (3) at a healthcare system level. The review findings identified a need for further training for primary care professionals on cancer care, clarity in the delineation of primary care and oncologist roles (i.e. who does what), effective communication and engagement between primary and secondary care, and the provision of protocols and guidelines for follow-up care in cancer.

**Conclusions:**

Information sharing and communication between primary and secondary care must improve to meet the increasing demand for support for people living with and beyond cancer. Delivering integrated pathways between primary and secondary care will yield improvements in patient outcomes and health economic costs.

**Supplementary Information:**

The online version contains supplementary material available at 10.1007/s00520-023-08278-1.

## Introduction

An estimated 19.3 million people were diagnosed with cancer across the world in 2020 with a forecasted 27.5 million cases in 2040 [1]. Advances in cancer treatment, earlier diagnoses, and a growing and aging population has meant more people are living with and beyond cancer. The number of people living with and beyond cancer in the United Kingdom (UK) is expected to grow by around 1 million every decade by 2030 [2]. People living with and beyond cancer may face a range of complex physical, psychosocial, and practical consequences lasting for months or years following treatment [3]. They generally have poorer health and wellbeing compared to the general population, with increased use of healthcare resources [4]. Increasing survival rates and an ageing population living with multi-morbidity adds further complexity. In addition, staff shortages in primary and secondary care and the impact of COVID on procedural delays are challenging health systems globally and affecting the ability to meet patient needs [5].

The care needs of people living with and beyond cancer are often not being optimally met [6–8]. Traditional approaches in which cancer patients are managed and followed up in hospital are no longer sustainable [9, 10]. Healthcare systems often work in silos, inhibiting collaborative working and the sharing of information between primary and secondary sectors [11]. Primary care and secondary care typically have separate information systems, performance indicators, and payment models, creating organisational barriers [12].

Effective integration of primary and secondary care services is important for ensuring consistent and comprehensive cancer care is delivered for patients [13, 14]. Integrated care is ‘…an organising principle for care delivery that aims to improve patient care and experience through improved coordination of services provided’ (p. 3) [15]. Key dimensions of integrated care focus on patient-centredness, multidisciplinary collaboration, and optimal care coordination [16]. While examples exist of integrated cancer care, supported by national recommendations [17, 18], these have not been widely adopted nor are there clear guidelines for how to implement this into existing healthcare systems. Improving integration of healthcare is an important policy driver for health systems globally [19]. In England, a recent Health and Care Act (2022) [20] formalised integrated care systems as legal bodies with statutory powers and responsibility to deliver multi-sectoral integrated care to better meet the needs of local health economies.

This scoping review answers the question: What are the facilitators and barriers to providing integrated care in cancer? The purpose of the review is to generate recommendations for clinical practice and health policy to support the implementation of effective integrated care for people living with and beyond cancer.

## Materials and methods

### Search methods for identification of studies

A scoping review was undertaken. Scoping reviews typically address broad research questions and include studies with different designs [21], examine a wide range of evidence, ensuring the breadth and depth of literature related to a particular topic is captured [22].

### Eligibility criteria

Included papers were published between 2009 and 2022. The year 2009 was chosen as a cut off owing to a series of key documents on cancer integration being published in this year. The review included papers in English that described the delivery of integrated primary and secondary cancer care. Quantitative, qualitative, mixed method studies, literature reviews, and policy documents were included. Non-English papers, conference articles, abstracts, and editorials were excluded.

### Electronic searches

Search terms focused on (a) cancer and (b) integrated care. The integrated care search strategy used an adapted search string from the Integrated Care Foundation [23] (see online resource 1). Ovid Medline was selected as the most relevant electronic database. Reference lists of included papers were searched for further relevant papers. The Kings Fund librarian team advised on searching their electronic database catalogue for grey literature. Search results were exported into EndNote V20.0, and duplicates were removed.

### Study selection

KL screened titles and abstracts of all search results. NC and KL screened full texts independently. Disagreement regarding inclusion was resolved through consensus decision with a third reviewer (DW).

### Assessment of quality

We assessed quality using Hawker et al.’s assessment form [24], appropriate for different paradigms. This form supported data extraction. No studies were excluded based on quality.

### Data extraction and management

KL and NC extracted data relevant to the research question independently, including author, publication year, study location, study aims, study design, study population, and outcomes (barriers and facilitators to integrated care, effectiveness of intervention (if applicable), and translation into clinical practice.

### Data synthesis

NC led a thematic synthesis, using the extracted data to generate themes [25]. The research team revised and refined themes iteratively to ensure plausibility and credibility.

Consideration of *micro* (e.g. individual experiences such as patient-healthcare professional interactions), *meso* (e.g. organisational aspects such as information systems between a primary care practice and hospital), and *macro* (e.g. organizing responses to structural and social determinates of health at the population level such as health policy) level factors informed the thematic synthesis [26]. These levels are interconnected and therefore may overlap.

## Results

Seventy-six potentially relevant research papers were identified, and duplicates and papers not meeting inclusion criteria were removed. Reasons for exclusion were insufficient data to answer the research question and studies focusing on intra- not inter-organisational integration of care.

Thirty-two articles were included (Table 1) (see online resource 2 for further details of included papers).

**Table 1:** Details of papers included in the scoping review

Study design	Number
Literature reviews	12
Qualitative research studies	10
Cross-sectional surveys	5
Mixed method studies	2
Randomised controlled trial	1
Non-randomised controlled trial	1
Literature review/interviews	1
Country	
Multiple countries	13
United Kingdom	7
United States of America	5
Australia	3
Canada	2
Denmark	1
Netherlands	1
Participant type	
Patients only	10
Healthcare professionals only	8
Patients and healthcare professionals	7
Patients and family caregivers	2
Patients, family caregivers, and healthcare professionals	3
Specific cancer service	2

**Fig. 1:**
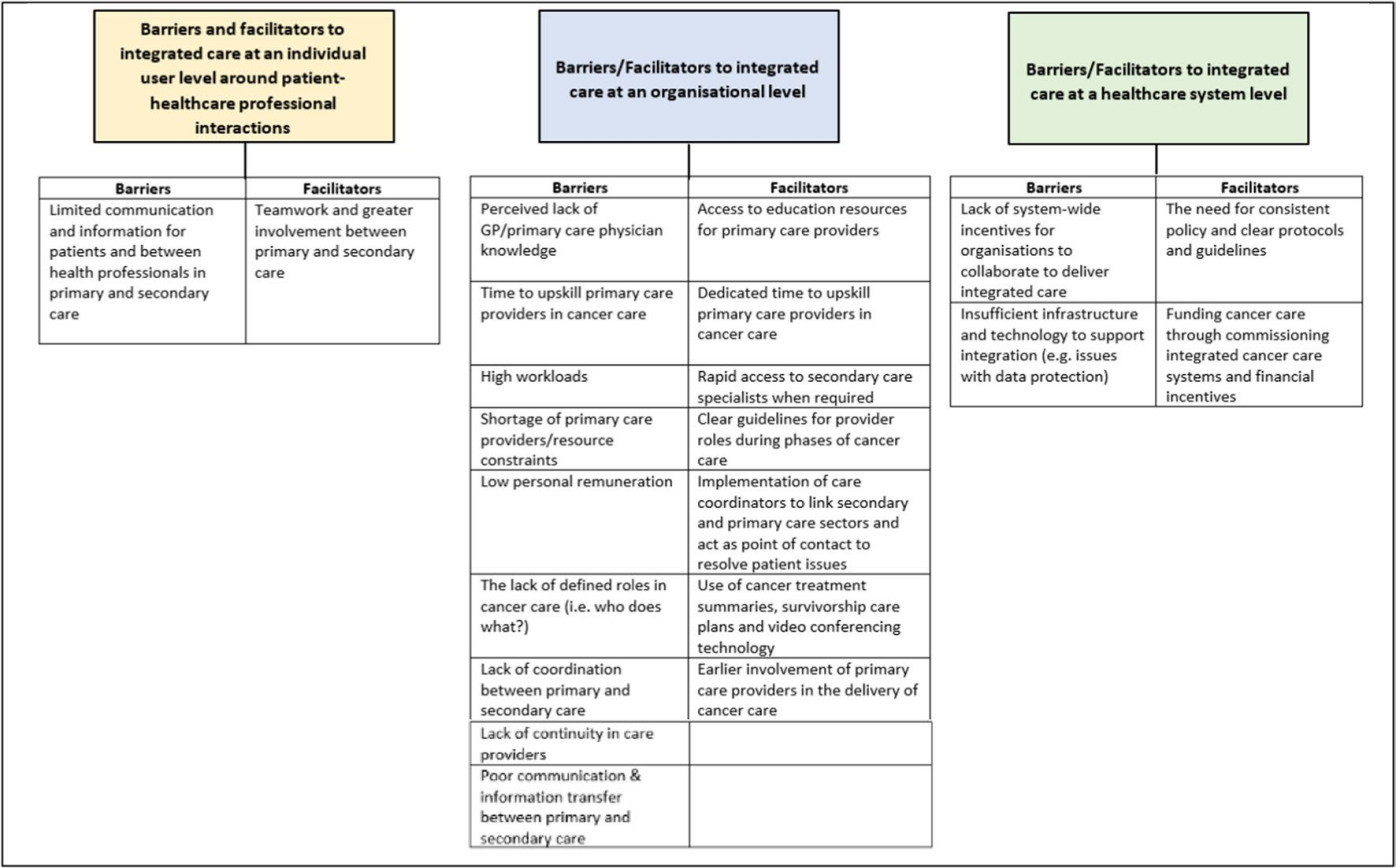
Overview of barriers/facilitators to integrated cancer care

Three key themes were identified on the barriers and facilitators to integrated cancer care (Fig. 1):

1. At an individual user level around patient-healthcare professional interactions
2. At an organisational level
3. At a healthcare system level

## Barriers and facilitators to integrated care at an individual user level around patient-healthcare professional interactions

### The quality of individual relationships between primary and secondary care and patients

#### Barriers

Limited communication between primary and secondary healthcare professionals is a significant barrier to integrated cancer care [27–35]. A study [36] comparing primary care providers’ and oncologist’s attitudes and practice regarding colorectal cancer survivors reported that most oncologists (67%) rarely or never provide a care plan summarising cancer treatments and surveillance recommendations for survivors. Moreover, over half of oncologists (59%) do not discuss with primary care providers who will follow up patients regarding cancer and other health issues. Lack of communication between primary and secondary care can undermine patients’ trust in healthcare providers and can compromise the delivery of high-quality care [36].

#### Facilitators

Effective working relationships between patient and primary care providers, patient and oncologists, and between primary and secondary care professionals are pivotal in providing integrated care, encouraging continuity and clarity about patients’ needs. One study reported mutual support for decision making, sharing thoughts regarding treatment or potential side effects between primary care providers and oncologists and acknowledging each other’s expertise aided good working relationships [34]. Involving primary care providers in multidisciplinary team meetings also provides an opportunity for developing relationships between primary and secondary care [34, 37]. Expanding multidisciplinary teams to include pharmacists with oncology training to advise on monitoring strategies based on patients’ treatment regimens could also facilitate integrated care [38].

Teamwork between primary and secondary care is also important in delivering integrated care and can aid the continuity of care, patient satisfaction, and information exchange [39]. Greater involvement of primary care and district nursing teams has been shown to be an effective means of distributing workload across primary and secondary care [40]. A systematic assessment of patient’s holistic needs, such as the ‘Holistic Needs Assessment’ in the UK, may help identify where other health professionals could support care for the patient [41].

## Barriers and facilitators to integrated care at an organisational level

### Skills, knowledge, and training of primary healthcare providers

#### Barriers

One of the most commonly reported barriers to integrated cancer care was a lack of clinical knowledge, training, and skills regarding cancer amongst primary care providers [13, 29–33, 35–38, 42–48]. This was reported by secondary care clinicians, patients, and primary care providers themselves. Areas where more knowledge was required included new cancer treatments, management of treatment side effects [9, 44], and follow-up requirements once treatment had ended (e.g. type, frequency, and duration of follow-up testing) [33]. Other areas included cancer screening, genetic testing, cancer survivorship issues [30, 37], and adverse event monitoring, assessing, and managing symptoms in the context of a cancer diagnosis were also cited as a learning need [33, 38, 44]. Reasons for a lack of knowledge and training in primary cancer care included inflexibility of health service career pathways and a lack of professional development opportunities [49].

One cross-sectional survey also reported primary care providers’ lack of understanding of referral processes to specialist cancer programmes [46]. Information about where a patient should seek help regarding health issues, which health professional to contact, and what diagnostic testing to have in place is generally not well understood by primary care providers, which is important if they are to remain involved in the care of their patient throughout the cancer trajectory [46].

Although primary care providers viewed themselves as valuable providers of survivorship care in cancer, they felt underprepared to perform the tasks needed for this role [38]. Oncologists appeared to concur with this view. For example, in one study, 54% of oncologists reported lacking confidence in primary care providers’ skills to provide follow-up care for the effects of cancer and its treatment [36]. Several studies found patients lacked confidence in their primary care providers’ knowledge and skills in cancer follow-up care [42, 50]. One qualitative study reported patients feeling let down by perceived diagnostic delays which may have led to late cancer diagnosis. This had implications for ongoing relationships with primary care providers and patients’ willingness to use primary care [42].

#### Facilitators

Facilitators to providing integrated care included relevant and clear information, cancer education resources for primary care providers, and rapid access to secondary care specialists when required [40, 43]. Building primary care providers’ skills in monitoring, assessing, and managing symptoms of cancer and follow-up care in cancer would also help facilitate effective cancer care integration [33, 38].

### Time for primary care providers to upskill in cancer care

#### Barriers

The literature commonly cited primary care provider’s high workload and, consequently, lack of time as barriers to participation in integrated care [13, 27, 33, 35, 37, 40, 43–45]. Overall shortages of primary care providers further hindered time to learn [37]. Resource constraints, in addition to low personal remuneration, were also barriers reported by primary care providers in one study on the provision of follow-up cancer care [33].

### Clarity of roles for healthcare professionals in cancer care in both primary and secondary care

#### Barriers

Lack of defined roles in cancer care, particularly the ambiguity of the primary care provider role, was commonly reported as a barrier to integrated cancer care [27, 29–31, 33–36, 38, 40, 45]. Meikeljohn’s [33] systematic review on primary care providers’ role in cancer care follow-up (2016) found the lack of clarity in the delineation of primary care provider and oncologist roles could lead to fragmentation of patient care between primary and secondary healthcare organisations. Another study emphasised the considerable ambiguity about which healthcare professional was primarily responsible for cancer-related follow-up, what should be done, and when, which resulted in a lack of care after cancer treatment, with patients falling between gaps between healthcare organisations [38].

Primary care providers may have differing attitudes about their role in providing cancer care. One study reported some primary care providers did not feel it was their role to provide cancer care [13]; another reported that most primary care providers saw it as an integral part of their role, although they doubted their ability to provide adequate information and support to patients [40]. A further study found primary care providers viewed their role as supporting patient’s health holistically and not specifically cancer [45].

#### Facilitators

Establishing clear guidelines for provider roles during various phases of cancer care was suggested as a means to maximize the skillsets of both primary and secondary care providers, improving the quality and coordination of cancer care [29]. Multidisciplinary video-based consultations between patients and healthcare professionals could be a successful way of clarifying tasks between the primary care provider and oncologist in a patient-centred way [34].

### Continuity and coordination of cancer care between primary and secondary care

#### Barriers

A lack of coordination between primary and secondary care [37] and, consequently, a lack of continuity of care for the patient [32] were barriers to integrated care. A scoping review exploring the relationship between integrated cancer care and patients’ experience reported that poor coordination could result in a duplication and breakdown in care, uncertainty around responsibility, delays in treatment and supportive care, and unmet patient care needs [39]. One qualitative study reported a lack of close working between healthcare professionals, with a lack of consultation and information exchange concerning the needs of patients and family caregivers. Consequently, patients and family caregivers felt their needs were inadequately supported. Indeed, patients and family caregivers reported being burdened with the task of ensuring information about their cancer treatment was communicated between healthcare professionals [51].

Logistical difficulties in coordinating care across different disciplinary and sectoral boundaries [34] and the lack of models promoting interdisciplinary cancer management have been cited as further barriers to integrated care. Different health professionals in cancer care tend to function through parallel (working in a common setting with each individual performing their job within their formally defined scope of practice) or consultative (expert advice given from one professional to another) models of care: both models are not well integrated in healthcare systems [52].

A further barrier to integrated cancer care is patients being seen by different professionals along the care pathway. Primary care providers can be disconnected from cancer treatment, meaning they might not be adequately involved in a patient’s follow-up care, hindering integrated care [37, 45]. A systematic review highlighted primary care providers feeling excluded from patients’ care during cancer treatment after diagnosis and were not clear when to re-establish contact with patients or what was expected from them [33, 40]. Research on continuity of care for follow-up and palliative care found primary care providers have insufficient time to build connections with secondary care and primary care providers, and patients do not receive the information they require from secondary care providers in a timely way [52].

#### Facilitators

Earlier involvement of primary care providers in the delivery of cancer care may facilitate integrated care [52]. Care coordinators can also facilitate integrated care by linking secondary and primary care, acting as a point of contact to resolve patient issues [41], chasing up appointments and results, and ensuring a smooth transition throughout the cancer care experience [49]. Care coordinators can improve the continuity of care and resolve confusion arising from the different responsibilities of diverse roles involved in cancer care provision [37]. A feasibility study implemented a model of integrated prostate cancer care involving online prostate cancer-specific holistic needs assessment (sHNA) and shared digital communication between patients and their health professionals. The study found that patient experience of care improved as nurses managed most needs identified by patients. The study also highlighted the value of nurses coordinating care through identifying and prioritising patient concerns and aiding decision-making regarding when to seek further medical care [53].

### Communication and information transfer between primary and secondary care

#### Barriers

Communication was identified as an important influence on providing effective integrated care [27–35]. Poor communication between primary and secondary care [48] can compromise high-quality surveillance care delivery [36], limit the ability to consult patients on issues related to their cancer [40], and can undermine relationships between health professionals, patients, and relatives [27]. A systematic review on primary care provider/secondary care cancer specialist relationships [29] reported the quality and timing of communication as a barrier to integrated care. This was particularly so when the communication from the secondary care cancer specialist to primary care providers had inadequate content. Equally, irrelevant content might result in a large volume of correspondence with primary care providers being unable to assess patients in a timely way. In several cases, primary care providers had to rely on updates from their patients [29].

Information transfer between primary and secondary care was frequently lacking because of separate IT infrastructures [28]. In one systematic review, primary care providers required additional information from oncologists regarding cancer treatments, follow-up plans, short and long-term side effects, suggested management, findings of investigations, and likely prognoses [44].

#### Facilitators

Strategies to improve communication and support integrated care delivery include the timely use of cancer treatment summaries and the development of survivorship care plans, which includes information about types of tests needed, frequency of check-ups, potential long-term late effects of the cancer treatments received, and suggestions for healthy living [9]. The use of videoconferencing technologies to connect primary care providers and specialists may also facilitate communication [30, 33, 46]. The use of tumour boards (groups of health professionals with different specialities discussing cancer cases and sharing knowledge) has also been shown to help engage geographically remote health professionals in collaborative care planning and delivery [52]. However, it is recognised that implementing inter-professional collaboration requires a significant change in culture [52].

Electronic records can be valuable in integrating follow-up care with survivors, supporting individuals and primary care providers through keeping schedules, facilitating communication, and promoting information access for survivors [31]. Shared data management systems through shared/integrated e-health records [41, 52] and enhanced communication between primary care providers and specialists [52] can facilitate care coordination and communication.

## Barriers/facilitators to integrated care at a healthcare system level

### Policy and guidelines

#### Barriers

There are few system-wide incentives for organisations to collaborate to deliver integrated care for people across the cancer journey. National and local healthcare priorities may fluctuate with shifting political agendas. Health system barriers to integration between primary and secondary services include insufficient infrastructure and technology, complicated by issues such as data protection [27].

#### Facilitators

A qualitative study exploring the views of cancer survivors, oncologists, and primary care providers about the primary care role in long-term cancer follow-up care reported the need for specific protocols to assist primary care providers in providing optimal care and as a safety net for recurrence or other serious events. Patient-specific follow-up protocols and plans were required, written by cancer specialists. Although overall responsibility for patients should remain with cancer specialists, routine elements of follow-up care could be performed by primary care providers [45]. The development of guidelines and detailed care pathways to ensure all patient needs are addressed within follow-up can facilitate integrated care [43].

### Funding of cancer care

#### Barriers

The way in which cancer care is funded, commissioned, and delivered means that services may not be aligned to individual needs [49]. For example, despite the introduction of integrated care systems in England, the commissioning of cancer services continues to be divided across multiple organisations, with primary and secondary care having separate funding models [28].

#### Facilitators

Financial incentives have been reported as a key element of success for care integration and patient outcomes [46]. One study [43] exploring the views of health professionals on the role of primary care in cancer follow-up reported that primary care providers stressed the importance of receiving financial remuneration to take on greater responsibility for cancer follow-up.

## Discussion

This review identifies and characterises key barriers and facilitators to providing integrated cancer care. Although a growing body of evidence supports integrated care models as effective ways to provide care for people living with and beyond cancer [28], challenges remain in providing this at micro, meso, and macro levels across health systems. Findings revealed the need for further training for primary care providers, clearly defined roles for healthcare professionals, effective communication and engagement between primary and secondary care, and the provision of protocols and guidelines for follow-up care in cancer. These findings resonate with those of a review of integrated primary and specialist cancer care [35]. Our review builds on the existing evidence of factors that enable or hinder integration of cancer care, for example, through the identification of other factors that facilitate integrated care at an organisational level, such as the implementation of care coordinators and use of videoconferencing technology which are relevant to all areas of cancer care, including follow-up.

A commonly reported factor in this review, which limits care integration, was a lack of training and opportunities for primary care providers to provide follow-up support to people with cancer. It is recognised that primary care providers play an important part in providing integrated personalised care and that rapid access to acute care and training opportunities will facilitate this [54]. Primary care providers are keen to engage in training opportunities to upskill in cancer, including case-based and experiential learning, short seminars, training resources, and e-learning programmes [55]. Including primary care in cancer follow-up has been linked to economic benefits. For example, primary care providers-led breast cancer follow-up has been shown to be cheaper than hospital-based follow-up with little difference in key outcomes [48]. Furthermore, primary care provision may reduce the number of hospital admission and days hospitalised in cancer patients over 70 years [56].

Although the majority of papers included in this review focus on the relationship between doctors in primary and secondary care, evidence shows the contribution nurses make to improved cancer patient experience through greater continuity of care and increased productivity [57, 58]. Nurse-led models in the management of cancer have shown improved efficiency, quality of care, and reduced costs over traditional follow-up [59]. Access to multidisciplinary survivorship care plans in which primary care nurses are actively involved in assessing physical, psychological, and social needs and supporting health education may help with efficient integration of personalised care. Survivorship care plans (SCPs) may include guidelines for monitoring and managing late effects of cancer treatment and/or recurrence, and information about diagnosis and lifestyle recommendations, and have been reported to improve coordination and continuity of survivorship care. E-health records systems accessible across primary and secondary care, to create, provide, and track SCPs could improve communication and coordination of care [60, 61]. Virtual delivery of healthcare services has become more commonplace in cancer pathways because of COVID-19. The use of video consultations may be a helpful resource to facilitate the sharing of information across diverse health sectors [62]. Assessment tools (such as the electronic holistic needs assessment in the UK) are increasingly digital, enabling assessment to be completed at home [49, 63]. This allows completed care plans to be shared between primary and secondary care digitally and thus enables personalised care and support planning to be embedded into the patient’s electronic record.

Integrated personalised care has been promoted for years, and various models of integration have been developed and tested, including the creation of multi-speciality community providers and primary acute care systems that focus on care pathways across primary, community, and acute settings. Innovative models or resourcing have sought to deliver multidisciplinary approaches to cancer care. In the UK, the National Health Services’ investment in a primary care network will support personalised care through further expansion of social prescribing, supporting digitised care and support planning for care home residents and shared decision making training [64]. However, the COVID-19 pandemic continues to challenge healthcare systems globally through increased staff shortages and therefore lack of protected time for healthcare professionals to train and upskill, as well as limited consultation time to deliver optimal cancer care. Subsequently, resources have been redirected, some screening services have closed, and the management of the backlog of procedures challenges the ability to provide continuous support [65]. Models of integrated care may need to be adapted to address the consequences of COVID and complex cancer cases. Furthermore, the UK model may not be easily transferable to other settings, for example, the implementation of the UK survivorship model in the USA may be challenging due to limited health care infrastructure, fragmented healthcare systems, and different survivor populations.

Collaboration is an integral component of integrated care and is characterised by good relationships at a local level. The value of a partnership mindset can be a successful approach in healthcare innovation, and working collaboratively across sectors can improve patient experience and outcomes. Ensuring patients are active partners rather than passive recipients of healthcare services and support is an essential component of effective partnerships between patients, services, and communities [66]. From the facilitators and barriers identified in this review, we suggest recommendations for practice and policy on the implementation of integrated care in cancer (see Table 2).

**Table 2:** Recommendations for policy and practice

Integrated care at an individual user level around patient-healthcare professional interactions
Include primary care providers as part of multidisciplinary team from the point of diagnosis
Enable an efficient line of communication between primary and secondary care providers- direct phone number or email address
Good communication between all health care providers and patients
Integrated care at an organisational level
Additional training for primary care providers
Facilitation of opportunities for health professionals from different disciplines to understand each other’s roles and professional identities to build trust, relationships, and joint ways of working
Clarify and agree on roles and responsibilities between health professionals in primary and secondary care team and communicate this to patients
Clear guidelines/protocols on management/follow-up care
Implementation of care coordinator/community link workers or navigator roles to aid scheduling appointments, advising patients, and facilitating communication between different healthcare providers
Patient centred focus through survivorship care plans, shared decision making, patient activation, setting care goals
Consider practical care models that facilitate primary care providers transition of role in survivorship cancer care
Integrated care at a healthcare system level
Foster partnerships and collaboration between primary and secondary care organisations
Establish clear protocols and guidelines for integrated cancer care
Consider incentivising GPs/primary care physicians in providing care for patients who have completed treatment
Standardization of survivorship care plans

### Limitations of review

The synthesis of a diverse range of evidence on integrated cancer care by a multidisciplinary team and a rigorous and systematic approach to literature searching are strengths of this paper. However, healthcare systems vary greatly across countries, and although international literature was included, the review evidence may not be universally applicable or easy to implement in all locations. There may also be other factors that challenge the implementation of integrated care for certain cancers that have not been considered in this paper.

## Conclusion

This review has synthesised qualitative and quantitative literature on the facilitators and barriers to providing integrated care in cancer. Fostering partnerships, collaboration, and innovative ways to share information between primary and secondary care will improve the provision of integrated cancer care.

### Supplementary Information

Below is the link to the electronic supplementary material.

Supplementary file1 (DOCX 12 KB)

Supplementary file2 (DOCX 25 KB)
