# Supplementary material for: Barriers and facilitators to integrated cancer care between primary and secondary care: a scoping review
Source: Support Care Cancer. 2024 Jan 22;32(2):120. doi: 10.1007/s00520-023-08278-1 (PMC10803398; doi:10.1007/s00520-023-08278-1)
Supplement: Supplementary file 1 — Supplementary file1 (DOCX 12 KB) [file 520_2023_8278_MOESM1_ESM.docx]

**Online Resource 1: Search strategy**

The database was searched using the following MeSH terms/subject heading between 2009 and 2022:

cancer.mp. [mp=title, abstract, original title, name of substance word, subject heading word, floating sub-heading word, keyword heading word, organism supplementary concept word, protocol supplementary concept word, rare disease supplementary concept word, unique identifier, synonyms]

AND

*Delivery of health care, integrated/ or Integrated care.mp,jw. or (integrated health*.mp. and og.xs.)

The Kings Fund Library catalogue was also searched for the grey literature using the subject headings: su:(integrated care AND cancer). Additional records were identified through references cited in included articles and relevant reviews.
