# Supplementary material for: Barriers and facilitators to integrated cancer care between primary and secondary care: a scoping review
Source: Support Care Cancer. 2024 Jan 22;32(2):120. doi: 10.1007/s00520-023-08278-1 (PMC10803398; doi:10.1007/s00520-023-08278-1)
Supplement: Supplementary file 2 — Supplementary file2 (DOCX 25 KB) [file 520_2023_8278_MOESM2_ESM.docx]

**Online Resource 2. List of included studies**

| **AUTHOR** | **YEAR** | **COUNTRY** | **STUDY DESIGN** | **STUDY AIM** | **POPULATION** |
| --- | --- | --- | --- | --- | --- |
| van Overveld | 2018 | The Netherlands | Qualitative (interviews) | To explore these needs and preferences, taking patients with head and neck cancer (HNC) as example, to adapt current integrated care to be more patient-centred. | 14 adult Dutch patients with head and neck cancer |
| Trabjerg | 2020 | Denmark | Qualitative (video consultations) | To explore the consultation structure, content, and task clarification when a GP and an oncologist are attending a video consultation with a patient with cancer. | 12 video consultations with adult patients with cancer |
| Sussman | 2017 | Canada | Cross sectional (survey) | To systematically examine the extent of integration of primary care physicians with a regional cancer program (RCP) for the care of cancer patients and to identify opportunities for integration to be improved, from the perspectives of PCPs | 473 primary care physicians across Canada |
| Dossett | 2017 | Multiple (USA, UK,  Canada, Australia, New Zealand, the Netherlands) | Systematic review | To describe the attributes of the relationship and communication between PCPs and cancer specialists. | 23 studies regarding primary care providers and cancer specialists |
| Perfors | 2021 | The Netherlands | Randomised Controlled Trial | To evaluate the effects of a time out consultation with a general practitioner on perceived shared decision making, information provision and self-efficacy | 154 adult patients with breast, colorectal, gynaecological or lung cancer or melanoma, and scheduled for curative treatment |
| Lisy | 2021 | Multiple (Australia, USA, UK, the Netherlands). | Systematic review | To explore facilitators and barriers to implementing shared cancer care. | Five papers included data from the perspectives  of HCPs exclusively, including three studies of general practitioners (GPs) only, four papers (three studies) included data from the perspective of patients only, and four from the perspective of both patients and HCPs. |
| Foglino | 2016 | Multiple (USA,  Canada, Australia, New Zealand, UK, Denmark, Spain, Sweden, Korea, Japan). | Scoping review | To describe the extent, scope and findings of the existing literature on the relationship between the integration of cancer services and patient experience. | 30 studies relating to integration of cancer services and patient experience. |
| den Herder-van der Eerden | 2017 | Multiple (Belgium, Germany, Hungary, the Netherlands and the United Kingdom) | Qualitative (longitudinal interviews) | To examine how relational, informational and management continuity of care are experienced by patients with advanced diseases and their family caregivers receiving care from several integrated palliative care initiatives in five European countries. | 152 patients with advanced disease (63% had cancer). 92 family caregivers. |
| Cortis | 2017 | Multiple (Australia, UK, Europe, North and South America) | A textual narrative literature synthesis | To examine how the conceptual  term *integrated care* has been applied within the cancer literature, to compare this to its use in primary care, and to discuss the relevance of this to future practice and research. | 38 studies with a direct focus on integrated care for patients with cancer. |
| Clarke | 2020 | UK | Non-randomised clustered controlled trial | To assess the feasibility of implementing a novel model of integrated prostate cancer care involving an online prostate cancer-specific holistic needs assessment (sHNA) and shared digital communication between patients and their healthcare professionals (HCPs). | Fourteen general practices and 41 adult males with prostate cancer. |
| Chubak | 2014 | USA | Cross-sectional survey 12 months after cancer diagnosis | To describe patient experiences and perspectives on the coordination between and the role of different providers in an integrated care system one year after cancer diagnosis. | 235 patients with cancer enrolled in previous randomised controlled trial. |
| Carmont | 2018 | Multiple (Australia, Denmark, the Netherlands, Canada, New Zealand) | Systematic review | To evaluate the effectiveness of interventions designed to engage GPs and specialist secondary services in integrated palliative care  To identify the personal, system and structural barriers and facilitators to integrated palliative care. | 17 studies involving adults receiving palliative care services through their GP, specialist hospital services or an integrated model of care were included. |
| Barnet | 2013 | Multiple (not stated) | Literature review and qualitative interviews | To review the literature pertaining to continuity of care in cancer treatment, with focus on consumer opinion pieces, and discuss how this may inform the planning of services. | Summary of the literature pertaining to continuity in cancer care with a focus on consumer opinion pieces and discusses how this may inform the planning of services.  Interviews with 12 patients and family members |
| Balasubramanian | 2018 | USA | Retrospective cohort study and a cross-sectional survey | To characterize colorectal cancer (CRC) surveillance patterns and correlates of receiving guideline- concordant CRC surveillance among under- and un-insured CRC survivors.  To compare primary care physicians’ and oncologists’ attitudes and practices regarding care of CRC cancer survivors in an integrated safety-net health system. | 205 patients diagnosed with Stage I–III colorectal cancer in an integrated system in the USA. |
| Macmillan | 2021 | UK | Literature review with patient vignettes | To take stock of how far the UK’s health and care services still need to go on integration for high- quality, personalised cancer care to be a reality for everyone. | Literature about adult patients with cancer. |
| Easley | 2017 | Canada | Qualitative (interviews) | To explore physician perspectives and contextual factors related to the coordination of cancer care and the role of family physicians. | 58 primary and cancer specialist health care providers from across Canada |
| Walters | 2015 | England | Cross-sectional (survey) | To determine the current practice and views of general practitioners in England regarding cancer survivorship care. | 500 English GPs. |
| Meiklejohn | 2016 | Multiple (Europe, UK, Australia, Canada, USA) | Systematic review | To explore the role of the general practitioners, family physicians and primary care physicians in the provision of follow up cancer care. | 25 quantitative and 33 qualitative articles were included focusing on patients' and GPs' perspectives of the GP role in follow-up cancer care. |
| Kendall | 2013 | UK | Mixed methods action research | To assess the feasibility of using a structured template to provide holistic follow up of patients in primary care from cancer diagnosis onwards. | 107 records for patients with a new diagnosis of cancer from 13 primary care teams45 interviews conducted with 6 patients with cancer and their carers and 29 health professionals (27 GPs and 2 practice nurses). |
| Adams | 2011 | UK | Qualitative (interviews and focus groups) | To describe: implementation of the QOF cancer care review patients’ experience of primary care over the first 3 years following a cancer diagnosis patients’ views on optimal care the views of primary care professionals regarding their cancer care. | 38 adults with 12 different cancer types were interviewed. 71 primary care team members took part in focus groups. |
| Lewis | 2009 | Multiple (UK, Norway, Sweden, Canada, Hong Kong) | Systematic review | To examine patients’ and healthcare professionals’ views about cancer follow up. | 19 studies were included examining patients/healthcare professionals views.  8 studies examined the views of healthcare professionals (four of which included GPs) 16 examined the views of patients. |
| Gagliardi | 2011 | Multiple (Australia, Belgium, Canada, Denmark, France, Norway, Spain, Sweden, UK, USA). | Literature review using a two phase meta-narrative approach | To describe conceptual models of collaboration and analyze how thy have been applied in the clinical management of cancer patients. | 22 studies on clinical management of cancer patients. |
| Lawrence | 2016 | Multiple (USA,  Canada, the Netherlands, Australia, Norway, France, UK, Germany, Italy, Ireland and Israel). | Systematic review | To summarise the available evidence on primary care physicians perspectives on their role within cancer care  To investigate PCP views of their role in providing care for cancer patients and survivors. | 35 studies representing the views of 10,941 primary care physicians. |
| Geramita | 2019 | USA | Cross-sectional survey | To examine primary care providers’ current knowledge, attitudes, beliefs and practices regarding their preparedness to provide survivorship care To explore predictors of confidence to identify groups of PCPs who may benefit from increased training and support. | 127 primary care providers (physicians, nurse practitioners, physician assistants). |
| Lawn | 2019 | Australia | Community forum | To explore Australian cancer survivors’ views on shared care. | 21 participants (11 cancer  survivors, 2 family caregivers, 8 clinicians and researchers). |
| Lewis | 2009 | Multiple (UK, Canada, Australia, Norway, Sweden, Denmark) | Systematic review | To compare the effectiveness and cost effectiveness of primary versus secondary care follow up of cancer patients, determine the effectiveness of the integration of primary care in routine hospital follow up and evaluate the impact of patient-initiated follow up on primary care. | 11 studies (10 RCTs and 1 non-randomised) (n=2,908 patients) and 2 ongoing studies comparing cancer follow-up in primary care with that in a secondary care setting or compared hospital follow-up with formal primary care involvement orpatient initiated follow-ups that reported primary care-related outcomes. |
| Watson | 2011 | UK | Qualitative (interviews) | To describe current practice in a sample of relevant healthcare professionals and to seek their views on the role of primary care in prostate cancer follow up. | 38 UK healthcare professionals consultant clinical (radiation) oncologists (n = 9), consultant urologists (n = 9), clinical nurse specialists (CNS) (n = 9), GPs (n = 9) and practice nurses (n = 2). |
| Ouwens | 2009 | Multiple (UK, USA,  Canada, Australia, Norway, Sweden, Denmark, the Netherlands) | Systematic review | To review integrated care interventions and their effects on the quality of care for patients with cancer. | 33 studies of adult patients with cancer focus on a rigorous evaluation of an integrated care intervention or of a programme with the aim of improving care for adult patients with cancer in hospital or in an out-patient setting. |
| Harley | 2011 | UK | Qualitative (interviews) | To improve understanding of chronic cancer from the perspective of patients and their informal carers. | 56 patients >12 months postdiagnosis of advanced cancer (breast (n=11); renal (n=11);  colorectal/gastrointestinal (n=12); gynaecological (n=12); and prostate (n=10)) |
| Hebdon | 2018 | USA | Qualitative (interviews) | To provide a multifaceted evaluation of survivorship care from the viewpoint of survivors, primary support individuals, primary care providers, oncology providers and registered nurses. | 20 participants included (5  patients, 6 relatives/friends, 9 healthcare professionals). |
| Johnson | 2018 | USA | Mixed methods using data from multiple sources:   1. historical data, 2. medical record review, (3) a mailed patient questionnaire, (4) 1:1 semi-structured telephone interviews with patients, and (5) 1:1 semi-structured interviews with staff members. | To evaluate whether the breast cancer survivorship program would improve the quality of care received by breast cancer survivors. | 85 patients completed  questionnaire. 20 patients interviewed. 20 staff members (7 registered nurses, 5 medical  doctors, 3 nurse practitioners, 2 integrative medicine practitioners, 1 pharmacist, 1 frontline staff member, and 1 radiation therapist). |
| Schütze | 2018 | Australia | Qualitative collective instrumental case study (interviews) | To investigate the attitudes and beliefs of cancer patients, their GPs and oncologists regarding the long-term follow-up care for patients with no current evidence of disease recurrence.  To explore the feasibility and acceptability of greater involvement of GPs in cancer follow up care in Australia. | 22 adult patients who had completed active treatment for breast and/or colorectal cancers and had no current evidence of disease  34 healthcare professionals (16 oncologists, 18 GPs). |
